# Mass Spectrometry-based PhyloProteomics (MSPP): A novel microbial typing Method

**DOI:** 10.1038/srep13431

**Published:** 2015-08-25

**Authors:** Andreas Erich Zautner, Wycliffe Omurwa Masanta, Michael Weig, Uwe Groß, Oliver Bader

**Affiliations:** 1 Institut für Medizinische Mikrobiologie, Universitätsmedizin Göttingen, Kreuzbergring 57, 37075 Göttingen, Germany

## Abstract

MALDI-TOF-MS of microorganisms, which identifies microbes based on masses of high abundant low molecular weight proteins, is rapidly advancing to become another standard method in clinical routine laboratory diagnostics. Allelic isoforms of these proteins result in varying masses of detectable biomarker ions. These variations give rise to a novel typing method for microorganisms named mass spectrometry-based phyloproteomics (MSPP). The base of MSPP is an amino acid sequence list of allelic isoforms caused by non-synonymous mutations in biomarker genes, which were detectable as mass shifts in an overlay of calibrated MALDI-TOF spectra. Thus, for each isolate a combination of amino acid sequences can be deduced from the scheme of recordable biomarker masses. Performing comparably to laborious multilocus and whole genome sequence typing (wgMLST)-approaches it is feasible to build phyloproteomic dendrograms using hierarchical cluster analysis. MSPP bears a high potential especially for identification of chromosomal localised virulence or antimicrobial resistance factors associated with evolutionary relatedness. In this study the principle of MSPP-typing was demonstrated on a *Campylobacter jejuni* ssp. *jejuni* isolate collection and MSPP was compared to MLST.

Typing of microorganisms below the species level is a common and necessary procedure to identify and track sources of nosocomial infections, to monitor migration of drug resistant strains, in surveillance of foodborne pathogens, or simply to investigate phylogenetic relatedness. The most commonly used method for this is MLST^1^ or one of its descendants like High-throughput MLST (HiMLST)^2^ and whole-genome MLST (wgMLST)^3^,^4^. Conventional MLST considers allelic nucleic acid variants of six to twelve housekeeping genes that are evenly distributed in the genome of the microbial species to be typed. These genes are sequenced using laborious classical PCR and subsequent Sanger sequencing of the amplicons^5^. The introduction of next generation sequencing (NGS) has led to procedures for sequencing of many isolates in parallel using isolate-specific multiplex identifiers (MID barcodes) and the expansion of MLST schemes to more loci^2^. However, the design of MLST schemes faces the problem to include a set of genes that are sufficient variable to reflect epidemiological relatedness. Thus, the decision on which and how many loci should be included depends largely on the particular microbial species and the specific epidemiological questions to be addressed. Therefore, MLST schemes consider only sufficient variable core genes and do not consider hypervariable, transposable gene loci and the whole genome sequence^6^. The two best-established NGS-based MLST schemes are ribosomal MLST (rMLST), which includes all 53 *rps* genes encoding bacterial ribosomal protein subunits^7^,^8^, and wgMLST using 1,643 defined gene loci in the case of *Campylobacter* species^3^. Both, wgMLST and rMLST databases are based on partially de novo assembled or fully annotated genome sequences, respectively, of a growing number of bacterial isolates^3^. Since these methods are still either very labor intensive or very expensive, typing is currently restricted to relatively small isolate cohorts and not universally available in clinical routine diagnostic contexts.

Over the past decade, intact cell MALDI-TOF mass spectrometry (ICMS) has become the current standard in clinical microbiological laboratories for species identification generating massive amounts of data on cellular proteomes of individual isolates^9^,^10^. ICMS uses protein fingerprints of cell lysates in the range of 2–20 kDa to identify a microbial species. In addition, ICMS has a potential to discriminate strains at the below-species level by accurately distinguishing biomarkers unique to a particular strain^11^. To a certain degree, we have previously shown that this peculiar characteristic can be exploited to distinguish *Salmonella enterica ssp. enterica* serovar *Typhi* from other clinically less-relevant *Salmonella enterica ssp. enterica* serotypes^12^ or, using a single biomarker ion (L32-M/CJ0330c) with different allelic isoforms, to discriminate (MLST-)sequence types ST22 and ST45 of *Campylobacter jejuni* ssp. *jejuni*^13^.

The application of ICMS in below-species differentiation is at its infancy. For this reason, the principal component analysis (PCA) hierarchical clustering of ICMS-spectra method^13^,^14^,^15^,^16^ and the hierarchical cluster analysis by unweighted pair group method with arithmetic mean (UPGMA) method for processing of the resulting mathematical matrix with a set of binary peak matching profiles^17^,^18^ are relied upon to convert variations in biomarker mass profiles into somewhat meaningful phyloproteomic relations. However, these clustering methods are very sensitive to peak intensity, mass spectrum quality, and microbial growth conditions making the inferred phyloproteomic relations difficult to reproduce. In contrast, diagnostic approaches to species and genus identification are mostly robust towards cultural conditions.

In this study, we use *C. jejuni* ssp. *jejuni* as a model clinical bacterium to develop a phyloproteomic typing scheme that combines the analysis of variable masses observed during ICMS with rMLST and wgMLST database-deduced isoform lists. This technique has the ability to perform below-species differentiation and whose mathematically inferred phyloproteomic relations are effortlessly reproducible. We name this technique – “mass spectrometry-based phyloproteomics (MSPP)”. Also, this technique unifies MLST and ICMS into one coherent and complementary below-species level differentiation process through mass spectrometric detection of allelic isoforms of selected biomarker ions with known genetic identity and the construction of a respective allelic isoform list. Phyloproteomic dendrograms are calculable by UPGMA analysis of the deducible amino acid sequences.

## Results

**Figure 1:**
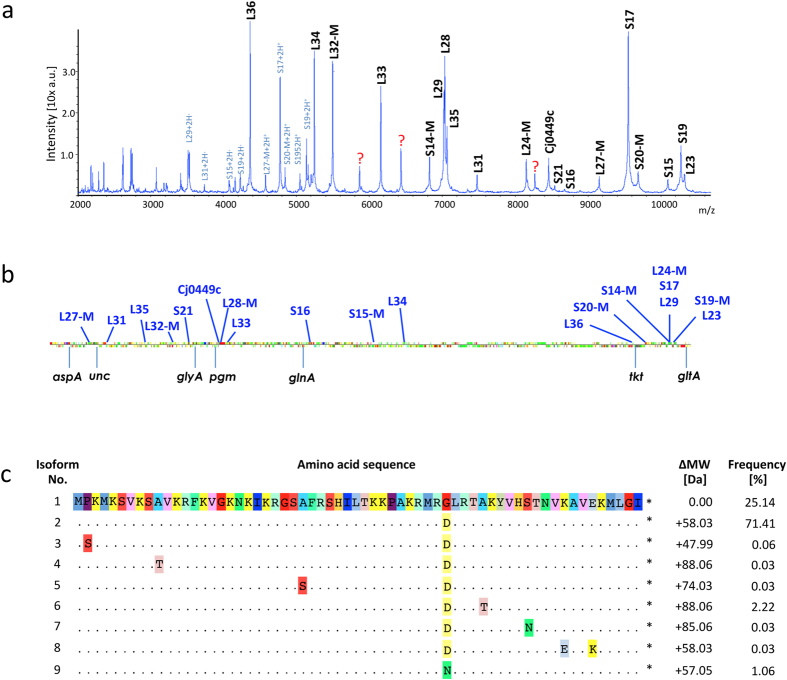
Establishing a MSPP-database. (**a**) ICMS spectrum of reference strain NCTC 11168, singularly charged biomarker ions that were identified by comparison of molecular masses with reference spectra are labeled black, multiple charged ions are labeled blue and three not yet identified biomarker ions are labeled with a red sign “?”. (**b**) Distribution of gene loci included in the *C. jejuni* ssp. *jejuni* MSPP (blue) and MLST (black) schemes on the chromosome of *C. jejuni* ssp. *jejuni* NCTC 11168. The MSPP loci are relatively evenly distributed throughout the genome. (**c**) Amino acid sequence-alignment and calculation of mass differences of RpmI/Cj0244 allelic isoforms from the rMLST database.

In contrast to whole-spectrum clustering approaches, where the presence or absence of single masses together with their abundance (peak intensity) are used to infer phylogeny, in MSPP only changes in mass assigned to one specific set of allelic isoforms of the same protein are considered. The workflow for establishing MSPP essentially is (I) to assign ICMS spectrum masses obtained from genome sequenced reference strains to protein coding genes, and (II) to compile a set of allelic isoforms of the assignable spectrum masses from larger sequence databases (Fig. 1).

**Figure 2:**
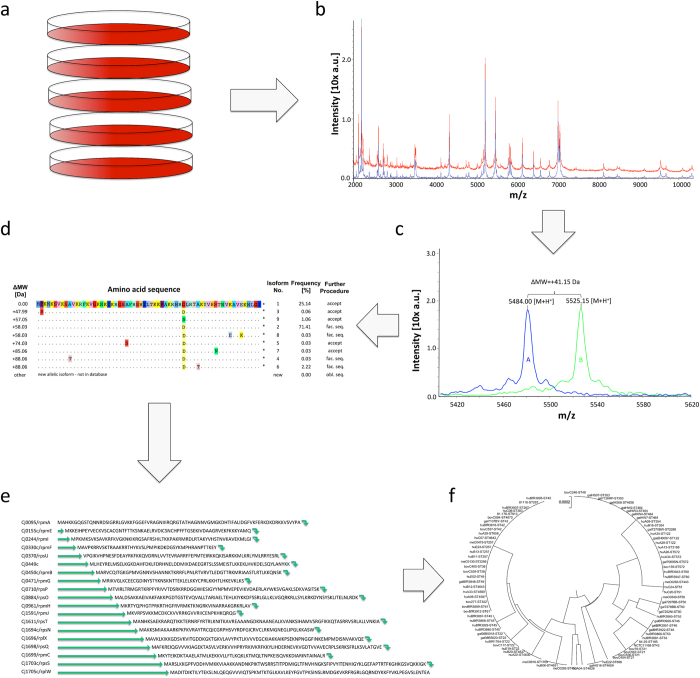
Schematic overview of MSPP workflow. (**a**) Microbial culture of all isolates included in a current typing setting. (**b**) Recording of ICMS mass spectra with subsequent calibration, smoothing and baseline subtraction. (**c**) Detection and quantification of mass differences of biomarker ions due to different allelic isoforms in particular strains. (**d**) Database search for allelic isoforms that correspond to the detected mass differences. (**e**) Joining of amino acid sequences from all biomarker ions included in the MSPP scheme (**f**) Construction of taxonomic dendrograms using the UPGMA method.

The MSPP typing procedure includes the following steps: (a) Culturing the microbial isolates to be typed, and (b) recording of ICMS spectra for each isolate (c) pre-processing and calibration of the recorded spectra, (d) measuring of mass shifts with reference to the genomic sequenced reference strain, (e) identification of particular allelic isoforms by matching the mass shifts with the database deduced isoform amino acid sequence set, (f) combination of amino acid sequences in the corresponding MSPP typing scheme and calculation of a phyloproteomic UPGMA-tree (Fig. 2).

*C. jejuni* ssp. *jejuni* was selected as a model organism for the validation of MSPP and the practical applicability of MSPP was demonstrated by typing a well characterised and MLST-typed *C. jejuni* ssp. *jejuni* isolate collection^13^,^19^,^20^,^21^.

### Identifying biomarker ions

Initially, ICMS of one (or if possible more) genome-sequenced reference strain(s) was performed and based on the masses predicted from the respective genome sequence MS biomarker ions were assigned to gene products corresponding to the measured mass. In the case of *C. jejuni* ssp. *jejuni*, the genome sequenced reference strain NCTC 11168^22^ was chosen as basis.

For assignment, the resulting spectrum was pre-processed by baseline subtraction and smoothing. Purified recombinant human insulin, which was added to the sample through spiking of the matrix solution, was used for single-point internal calibration. Recombinant insulin was chosen as internal calibrant because its mass did not mask any visible mass of *C. jejuni* ssp. *jejuni*. Importantly, if the respective singly charged mass was evident, multiple charged ions were eliminated from the list of potential biomarkers (Fig. 1a). Removal of *N*-terminal methionine being a major post-translational modification was taken into account with a mass difference of −131 Da^23^.

Based on the genome sequence of strain NCTC 11168 nineteen monoisotopic, singly charged biomarker masses within the range of 2000 to 11000 m/z could be presumptively matched to a specific locus with no more than 1 Da mass difference. These biomarkers were RpmA (9153.45 Da), RpmE (7476.68 Da), RpmI (7153.78 Da), RpmF (5496.45 Da), RpsU (8641.27 Da), RpmB (7064.27 Da), RpmG (6156.29 Da), RpsP (8678.14 Da), RpsO (10094.85 Da), RpmH (5244.28 Da), RpmJ (4364.39 Da), RpsT (9685.29 Da), RpsN (6825.27 Da), RplX (8151.75 Da), RpsQ (9549.39 Da), RpmC (7033.49 Da), RpsS (10322.08 Da), RplW (10567.35 Da) and a non-ribosomal protein Cj0449c (8458.60 Da). Three biomarker masses (5865.73 Da; 6435.34 Da; and 8255.21 Da) could not be unequivocally matched to a specific locus (Fig. 1a). In total, 7 of 21 ribosomal proteins of the 30 S subunit and 11 of 31 (RpmD/L30 is constitutively missing in *C. jejuni* ssp. *Jejuni*) ribosomal proteins of the 50 S subunit could be included in the MSPP scheme. As a result, the *C. jejuni* ssp. *jejuni* MSPP scheme comprised of 19 proteins, which are distributed throughout the genome of strain NCTC 11168 in a similar fashion as exhibited by the seven established MLST markers (Fig. 1b).

### Compiling an allelic isoforms list

Initially, a list of potential allelic isoforms for all masses of the *C. jejuni* ssp*. jejuni*-specific MSPP scheme was compiled (Fig. 1c, Supplementary Table 2). Here, we benefited from the broad spectrum of sequences deposited in the wgMLST database^3^ and rMLST database^7^. At the time of this analysis, 3477 *C. jejuni* ssp. *jejuni* genome sequences had been deposited in the rMLST database and 2364 in the wgMLST database.

The respective sequences deposited for each potential marker were translated to amino acids and aligned. The protein mass of each individual isoform as well as the mass change of each isoform with respect to the reference strain was calculated. Between 3 and 22 isoforms could be identified within the rMLST and wgMLST databases for each mass that was included in the MSPP scheme. These were represented with different frequencies, ranging from >98% to single occurrences. In the case of single occurrence in the database, a sequencing error cannot be excluded. However, for most biomarkers at least two major isoforms were found, suggesting that this set of masses could indeed serve as phylogenetic discriminators.

### Typing a microbial isolate collection

**Figure 3:**
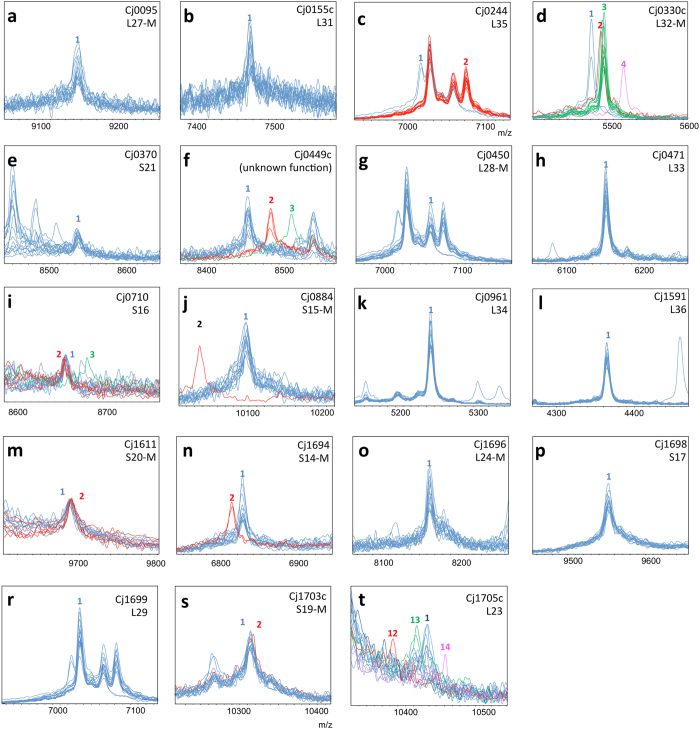
Mass spectra of *C. jejuni* ssp. *jejuni* MSPP Biomarkers with identifiable coding genes. (**a–u**) Representative spectra for the different MSPP types were overlaid to visualize mass differences between the isoforms observed in our test set. X-Axis: mass[Da]∙charge^−1^ ratio, scale 200 Da. Y-Axis: intensity [arbitrary units], spectra were individually adjusted to similar noise level for better visualization of low-intensity peaks. Color code: reference strain NCTC 11168 isoform (blue); other isoforms (red, green, and purple). De-methioninated isoforms are indicated by “-M” following the ribosomal subunit name (panels **a**,**d**,**g**,**j**,**n**,**o**,**p** and **t**). Numbering of isoforms refers to Supplementary Table 2.

To validate the *C. jejuni* ssp. *jejuni*-MSPP, we screened a collection of 96^19^,^20^ clinical isolates and four genome sequenced strains NCTC 11168, NCTC 11828 (81116), 81–1763, and 84-25 for mass shifts of the 19 biomarkers as listed above (Fig. 3). The isolates were chosen in such a way that all major MLST groups were represented. All isolates were cultured on Columbia blood agar and were incubated overnight at 42 °C in a semi anaerobic atmosphere. The following day ICMS was performed by both, the regular smear and formic acid/acetonitrile extraction methods.

### Recording and pre-processing of ICMS spectra, measuring of mass shifts and identification of allelic isoforms

ICMS was performed as outlined for the initial analysis on strain NCTC 11168 (Fig. 2b). Mass shifts were measured with reference to this reference strain (Fig. 2c and Fig. 3). A particular allelic isoform was identified by comparison of the mass shift with the isoform amino acid sequence list (Supplementary Table 2 and Fig. 2d, respectively). Only in the case of isoforms with the same mass difference, i.e. with the same amino acid substitutions but at different positions of the amino acid sequence additional DNA sequencing was necessary (e.g. Fig. 2d; isoforms no. 4 and 6).

Within this study population we detected 2 different isoforms for Cj0244/RpmI, 4 for Cj0330c/RpmF, 4 for Cj0449c, 3 for Cj0710/RpsP, 2 for Cj0884/RpsO, 2 for Cj1611/RpsT, 2 for Cj1694c/RpsN, 2 for Cj1703c/RpsS and 4 for Cj1705c/RplW (Fig. 3). Only one allelic isoform corresponding to that present in reference strain NCTC 11168 was detected for the remaining ten proteins marked as potential biomarkers in the ICMS spectra and included in the *C. jejuni* ssp. *jejuni* MSPP-scheme, namely, Cj0095/RpmA, Cj0155c/RpmE, Cj0370/RpsU, Cj0450c/RpmB, Cj0471/RpmG, Cj0961/RpmH, Cj1591/RpmJ, Cj1696/RplX, Cj1698/RpsQ, and Cj1699/RpmC. Sanger sequencing confirmed all novel isoforms detected by mass spectrometry, thus experimentally verifying the identity of nine of the 19 biomarkers.

### Calculation of a phyloproteomic UPGMA-tree

**Figure 4:**
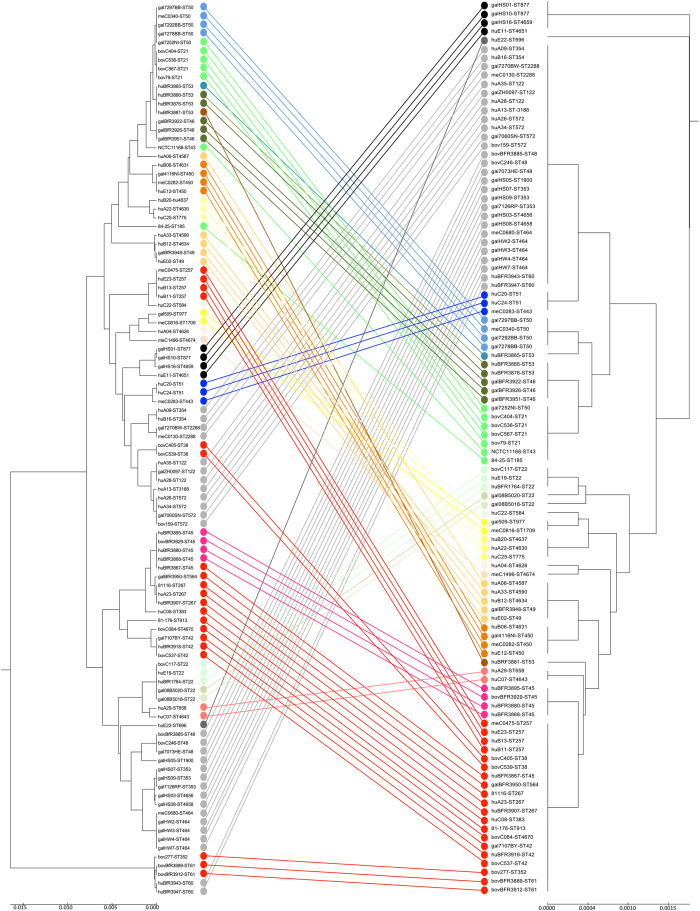
Comparison of MSPP- and MLST-based phyloproteomic trees. Both the seven loci MLST-based dendrogram (left) and the nineteen biomarker ion MSPP-based dendrogram (right) were constructed using the UPGMA-method. In both trees taxa arrangement is according to the implemented balanced shape function. The 22 different MSPP sequence types are represented by 22 different colours. Lines in one of the 22 colours connect the corresponding isolates in the different trees. The predominantly parallel lines illustrate the strong correspondence of the MSPP sequence types with the specific MSLT STs and/or STs.

In analogy to MLST clustering, the concatenated amino acid sequences of the identified isoforms were fused to one continuous sequence (Fig. 2e). This gave rise to different sequence types, which were used to infer the phylogeny by conventional clustering algorithms, UPGMA (Figs 2f and 4).

By combination of the amino acid sequences of the allelic isoforms present in our collection, 22 different MSPP sequence types were observed. The dendrogram constructed from the concatenated biomarker protein sequences showed a general concordance with the respective MLST phylogeny (Fig. 4). The *C. jejuni* ssp. *jejuni* MSPP scheme clearly discriminated isolates belonging to the MLST clonal complexes (CC) and MLST sequence types (ST) CC658/ST658, CC446/ST450, CC49/ST49, CC1034/ST977, CC22/ST22, CC45/ST45, CC21/ST22, CC206/ST46, CC21/ST53, CC21/ST50, CC443/ST443 and ST51, ST877 and related STs (no CC defined) respectively. Whereas CC283, CC42, CC257, CC48, and some CC61 isolates grouped together into one cluster, and CC48, CC353, ST464 (no CC defined), CC354, CC206/ST122, CC206/ST572, and CC61/ST60 grouped together into a second lager cluster (Fig. 4). In conclusion, 12 out of 23 relevant MLST CC/STs were sufficiently discriminated by the *C. jejuni* ssp. *jejuni* MSPP scheme, while only nine of the 19 potential biomarker masses showed varying allelic isoforms in our isolate set.

### PCA analysis of *Campylobacter jejuni ssp. jejuni* isolates

To exemplify the influence of different culture media and incubation temperatures for *C. jejuni* ssp. *jejuni*, ICMS-spectra-based PCA-dendrograms of 22 *C. jejuni* spp. *jejuni* isolates representing the 22 different MSPP sequence types were cultured for 24 hrs on (A) Columbia sheep blood agar plates, Mueller Hinton horse blood agar, and Oxoid Campylobacter agar supplemented with lysed horse blood at 42 °C; on (B) 2 different batches of Columbia sheep blood agar plates each at 42 °C; and on (C) 2 different batches of Columbia sheep blood agar plates (same lot) at 42 °C and 37 °C. The mass spectra of all 110 samples have been recorded and PCA cluster analyses have been performed. According to the phylogenetic identity it has been expected that despite of the different culture conditions identical isolates group as couples or triplets. Instead of the grouping as couples and triplets the isolates predominantly form subclusters according to their cultural conditions. In summary, the different culture conditions significantly interfered with the phylogenetic identity (see Supplementary Figure 1A–C).

## Discussion

Recently different studies identified particular individual biomarker masses from ICMS spectra which were present or absent in a particular subset of strains, for example biomarkers for differentiation of the five major methicillin resistant *Staphylococcus aureus* (MRSA) clonal complexes corresponding to the five major pulsed field gel electrophoresis (PFGE) MRSA types^24^,^25^. Additionally a set of biomarkers was identified that was able to distinguish between methicillin-resistant and vancomycin-intermediate *S. aureus* (VISA) strains, vancomycin-susceptible *S. aureus* (VSSA) strains, as well as between staphylococcal cassette chromosome *mec* (SCC*mec*) type IV & V and SCC*mec* type I–III isolates^26^. Furthermore singular biomarkers for the differentiation of specific isolate groups of *Clostridium difficile*^27^, *Salmonella enterica*^12^, and *E. coli*^15^,^16^,^28^ have been described. This absence or presence of biomarker ions is most likely due to absence or presence of non-ubiquitous genes. Because of the mostly unknown identity of the biomarker masses in these pioneering studies it is likely that biomarker mass shifts have been misinterpreted as absence or presence of biomarker ions.

Also, shifts in biomarker masses between subspecies and strains have been described and found to be the result of non-synonymous mutations in the encoding gene and to correlate with the phylogenetic relatedness of strains^13^,^23^,^29^. Fagerquist and co-wokers demonstrated that biomarker mass shifts can be used to discriminate between *C. jejuni* ssp. *jejuni* and *C. jejuni* ssp. *doylei*^23^,^29^. While we have previously identified a biomarker ion that exists in different allelic isoforms resulting in a mass shift specific for MLST-sequence types ST22 and ST45 of *Campylobacter jejuni* ssp*. jejuni*^13^.

There are crude and simple mathematical tools transforming these variations in protein fingerprints and particular biomarker masses without identification of specific biomarker ions into phyloproteomic relations. An integrated and therefore often-used approach is principal component analysis (PCA) hierarchical clustering of ICMS-spectra, e.g. as implemented within the MALDI Biotyper Software (Bruker Daltonics, Bremen, Germany)^13^,^14^,^15^,^16^. However, PCA clustering is very sensitive to culture conditions as it takes not only peak masses, but also peak intensities into consideration. Therefore PCA results are often difficult to reproduce^13^. Another method to visualize phyloproteomic relations is the processing of a mathematical matrix with a set of binary peak matching profiles using the UPGMA method^17^,^18^. Alternatively peak list-peak list similarities can be represented as a dendrogram applying the single linkage cluster algorithm^30^. The advantage of these clustering methods is that they are not necessarily based on an identification of the considered biomarker masses. However, the reproducibility of all these methods suffers from poor spectra, what can be problematic if particular peaks are not recordable while the quality of the overall mass spectrum seems well. Since these methods do not distinguish sufficiently between peak shifts and non-ubiquitous biomarker masses, phyloproteomic relatedness may vary significantly with every independent recording of mass spectra. In MSPP this is circumvented by translation of mass shifts of biomarker into amino acid sequences of the respective allelic isoform. Low-quality spectra are identified where masses included in the scheme are absent and can be excluded from the analysis. Such datasets can easily be combined with sets derived from larger MLST databases such as wgMLST. The reliability of constructed dendrograms and deduced phyloproteomic and therewith phylogenetic relatedness is much higher and more clearly reproducible in comparison to PCA clustering of masses and their intensities. In contrast to PCA clustering, MSPP-based UPGMA-clustering is insensitive to culture conditions and results obtained from smear preparations are comparable to those of formic acid/acetonitrile extraction.

The principle of MSPP was applied in this study on a well-characterised *C. jejuni* ssp. *jejuni* isolate collection^19^,^20^. The MSPP scheme presented for *C. jejuni* ssp. *jejuni* combines nineteen different biomarker ions that could be assigned mostly to ribosomal proteins. Applying the scheme to our collection sufficiently discriminated 12 out of 23 relevant MLST CC/ST. To increase the discriminatory power of the MSPP-technique, future studies should be directed towards widening the recordable spectral mass range above the current limit of approximately 11000 m/z and assigning identities to so far unidentified biomarker ions. In the case of *C. jejuni* ssp. *jejuni*, three additional detectable biomarker ions show mass shifts but since they could not be associated with a particular gene locus, they could not be included in the MSPP scheme yet (denoted by question marks in Fig. 1a). Nevertheless, shifts showed specificity for some of the remaining MLST CCs/STs clustering together in the above-mentioned two larger MSPP-complexes, making them interesting biomarkers. Identification of such orphan biomasses will complement the *C. jejuni* ssp. *jejuni* MSPP scheme^28^,^31^.

In addition to its intrinsic reproducibility, other advantages include (i) its relatively low costs, even when analysing large numbers of isolates in parallel and (ii) the possibility to combine MSPP with classical DNA sequence based MLST (using primers for the loci included in the MSPP scheme) or wgMLST approaches. Such DNA sequence based methodologies must simply include the same loci/allelic isoforms included in the MSPP scheme. A r/wgMLST database associated mass spectra database would be useful. Even a related, meaning a r/wgMLST based nomenclature could be used for MSPP. It would be expedient to designate the MSPP isoforms with the lowest numerical identifier of the corresponding synonymous DNA allele sequence used in MLST.

MSPP will likely be applicable to a variety of clinically highly important problems, such as delineation of the major methicillin resistant *S. aureus* (MRSA) clonal complexes^24^,^25^, distinction between MRSA and MSSA, VISA and VSSA, and between different SCC*mec* types^26^. Furthermore, we have generated preliminary data to show that MSPP can successfully be applied in the subtyping of *Staphylococcus aureus*, *Escherichia coli*, *Clostridium difficile*, *Campylobacter coli*, and *Salmonella enterica ssp. enterica*. Depending on the accessibility to a significant number of whole genome shotgun sequences other bacterial and also fungal species will follow. Since the presence of many virulence and resistance factors are associated with phylogenetic/phyloproteomic subgroups, MSPP has the potential to advance to a useful tool predicting antibiotic susceptibility patterns and highly virulent microbial populations. Although porting MSPP to further species and clinical problems of high relevance will require significant effort, automated procedures unlocking the trove of data already available from spectra acquired during clinical routine diagnostics could significantly improve tracking of nosocomial microbes and on-the-fly calculation of likelihoods for drug resistance phenotypes.

## Materials and Methods

### Campylobacter jejuni ssp. jejuni strains

Four genome-sequenced *C. jejuni* ssp. *jejuni* strains NCTC 11168^22^, NCTC 11828 (81116)^32^, 81–176^33^, and 84-25 (Fouts, D. and Nelson, K.; unpublished, Gi01264) and 96 previously characterised *C. jejuni* ssp. *jejuni* isolates^19^,^20^ were selected for Mass Spectrometry-based PhyloProteomics (MSPP) analyses. The 96 isolates include: 43 isolates of human, 30 of chicken, 16 of bovine, and 7 of turkey origin, which had previously been genetically characterised for 16 different genetic markers^19^,^20^ and were typed by MLST^21^. NCTC 11168, NCTC 11828 (81116), 81–176, and 84-25 were obtained from Leibniz Institute - DSMZ German Collection of Microorganisms and Cell Cultures, Braunschweig, Germany. Avian and bovine isolates were provided by the German *Campylobacter* Reference Center of the *Bundesinstitut für Risikobewertung* (Federal Institute for Risk Assessment) in Berlin, Germany. Human isolates originated from stool samples of suspected campylobacteriosis patients treated at the University Medical Center Göttingen (Germany).

### *Campylobacter jejuni* ssp. *jejuni* culture conditions

*C. jejuni* ssp. *jejuni* isolates were stored as cryobank stocks (Mast Diagnostica, Reinfeld, Germany) at −80 °C. For usage in this study, they were cultured as one batch on Columbia agar base (Merck, Darmstadt, Germany) supplemented with 5% sheep blood (Oxoid Deutschland GmbH, Wesel, Germany) and incubated overnight at 42 °C under microaerophilic conditions (5% O_2_, 10% CO_2_, 85% N_2_). Experiments were performed under biosafety level 2 conditions.

### Preparation of an human insulin-containing matrix solution

For preparation, the α-cyano-4-hydroxy-cinnamic acid (HCCA) matrix purified matrix substance (Bruker Daltonics, Bremen, Germany) was dissolved in standard solvent: acetonitrile 50%, water 47.5% and trifluoroacetic acid 2.5% (Sigma-Aldrich, Taufkirchen, Germany) to a final concentration of 10 mg HCCA/mL. Purified recombinant human insulin (Sigma-Aldrich, Taufkirchen, Germany) was added as internal calibrant to the HCCA-matrix solution. Human insulin was dissolved to a final concentration of 10 pg/μL in 50% aqueous acetonitrile. The exact mass of the insulin peak was determined experimentally by mixing with Bruker Test Standard: m/z = 5806.1. The insulin peak was used for internal calibration of all *C. jejuni* ssp. *jejuni* mass spectra because it did not coincide with any other observed biomarker masses. Using an internal calibrant substantially increased the precision when determining variations of biomarker masses. Using this approach, we were able to detect differences in mass of up to 1 Da.

### MALDI-TOF mass spectrometry

Samples for MALDI-TOF MS were prepared in duplicate either by smear preparation or extraction. Extracts were prepared by harvesting five colonies of an overnight agar plate culture, which were thoroughly suspended in 300 μL double-distilled water. Subsequently, 900 μL absolute ethanol was added and the suspension thorough mixed by multiple pipetting until the bacterial colonies were completely suspended. These suspensions were centrifuged at 13,000 × g for 1 minute, the supernatant was discarded, and the pellets were dried at room temperature for 10 minutes. Upon drying, the pellet was thoroughly re-suspended by vortexing in 50 μL of 70% formic acid. Subsequently, 50 μL of acetonitrile was added to each sample and mixed by pipetting up and down. The mixture was centrifuged at 13,000 × g for 2 min. 1 μL of the supernatant was transferred onto a sample position on a polished steel MALDI target plate and left to dry for 5 minutes at room temperature. After drying each sample position was overlaid with 1 μL of HCCA matrix containing the internal calibrant and left to dry at room temperature. After which the matrix the samples were ready for MS-analysis^34^.

ICMS was done by standard procedures recommended for the MALDI Biotyper system (Bruker Daltonics, Bremen, Germany). For analysis, 600 spectra from 2–20 kDa were gathered in 100-shots steps on an Autoflex III system and added up. Results with MALDI Biotyper identification score values ≥2.000 were considered correct.

### Identification of biomarker ions from mass spectra

Analyses of mass spectra were done using the standard algorithms implemented in FlexAnalysis (Bruker Daltonics, Bremen, Germany). Spectra were first internally calibrated to the spiked insulin peak (m/z = 5806.1), and subsequently pre-processed by baseline subtraction and smoothing. The theoretical monoisotopic molecular weight of the proteins corresponding to each open reading frame was deduced from the amino acid sequence using the molecular weight calculator tool at the ExPASy Bioinformatics Resource Portal (http://web.expasy.org/compute_pi/). Occasionally, ribosomal proteins of Enterobacteriaceae undergo posttranslational modification by proteolytic removal of the *N*-terminal methionine. Consequently, for each open reading frame two optional molecular weights had to been taken into consideration^35^. Identification of biomarker masses, that means assignment of a biomarker mass to a specific allelic isoform, was firstly, performed by matching the measured masses with the calculated masses from the NCTC 11168 reference genome. But if the biomarker mass in the spectrum of a particular clinical isolate did not clearly correspond to the mass calculated from the NCTC 11168 reference genome, biomarker mass identification was performed in the second instance by matching to calculated masses of entries of the ribosomal MLST (rMLST) database or the whole genome MLST (wgMLST) database, respectively. Lastly, if there were still no clear matches in biomarker mass, the spectrum was screened for peaks with a molecular weight difference with plausible amino acid exchange(s) that could potentially explain the mass shift (Supplementary Table 1). All allelic isoforms (Supplementary Table 2) were reconfirmed by Sanger sequencing (Seqlab, Göttingen, Germany) of amplicons produced by the suspected gene (Supplementary Figure 4). The particular primers are listed in Supplementary Table 3. All the PCR reactions were performed under the following condition: denaturation at 94  C for 30 sec; annealing at 55 °C for 30 sec; elongation at 72 °C for 30 sec. In all cases, the predicted amino acid exchanges were found encoded in the genes, which in turn served as proof for the peak identity.

### Phylogenetic and Phyloproteomic analysis

Biological sequence alignment editor - BioEdit (http://www.mbio.ncsu.edu/bioedit/bioedit.html)^36^ was used for translation and protein sequence alignment of the sequences obtained from the rMLST and wgMLST database to compile the list of amino acid sequences of all allelic isoforms of the 19 biomarkers included in the MSPP-scheme (Supplementary Table 2). BioEdit was also used for DNA and protein sequence trimming and alignment of the sequences resulting from confirmatory Sanger sequencing.

MEGA6 software was used for construction of a UPGMA-dendrogram (unweighted-pair group method using average linkages)^37^, and *C. jejuni* ssp. *jejuni* MLST website (http://pubmlst.org/campylobacter/) was consulted for designation of sequence types and clonal complexes^38^.

PCA-analyses were performed using the algorithms implemented into the MALDI Biotyper 3.0 software (both Bruker Daltonics, Bremen, Germany). Spectra were pre-processed by baseline subtraction and smoothing, for ICMS-spectra-based PCA hierarchical clustering distance measurement was set to ‘correlation’; the linkage algorithm to ‘average’.

### Proposed Nomenclature of Mass Spectrometry-based PhyloProteomics

The nomenclature to be used for MSPP can be deduced from that of extended MLST schemes, particularly, the rMLST scheme. Each unique m/z-value corresponds to one or more allelic isoforms matching to a particular amino acid sequence. Because of the degeneration of the genetic code, a particular amino acid sequence in turn can correspond to one or more allele sequence/-s deposited in the MLST database. For a specific allelic isoform detectable by MALDI-TOF MS, the MLST allele designation with the lowest numerical identifier should be used as MSPP isoform number. For example, the allele coding for the cj0449c isoform detectable at a mass of m/z = 8458.60 in strain NCTC 11168 has been assigned the allele number 1 in the MLST database and thus also the MSPP isoform number 1.

## Ethical Approval

Ethical clearance for the analysis was obtained from Ethics Committee of the University Medical Center Göttingen, Germany. No humans or animals were used for this study.

## Additional Information

**How to cite this article**: Zautner, A.E. *et al.* Mass Spectrometry-based PhyloProteomics (MSPP): A novel microbial typing Method. *Sci. Rep.* **5**, 13431; doi: 10.1038/srep13431 (2015).

## Supplementary Material

Supplementary Information
