# Supplementary material for: Mass Spectrometry-based PhyloProteomics (MSPP): A novel microbial typing Method
Source: Sci Rep. 2015 Aug 25;5:13431. doi: 10.1038/srep13431 (PMC4548220; doi:10.1038/srep13431)
Supplement: Supplementary Information [file srep13431-s1.pdf]

Supplementary Information for the manuscript:

Mass Spectrometry-based PhyloProteomics (MSPP): A novel microbial typing Method

Andreas Erich Zautner[1,§], Wycliffe Omurwa Masanta[1], Michael Weig[1], Uwe Groß[1], Oliver Bader[1]

[1] Institut für Medizinische Mikrobiologie, Universitätsmedizin Göttingen, Kreuzbergring 57, 37075 Göttingen, Germany

[§] Corresponding author: Andreas E. Zautner
Universitätsmedizin Göttingen
Institut für Medizinische Mikrobiologie
Kreuzbergring 57
D-37075 Göttingen, Germany
Phone: +49-551-398549
FAX: +49-551-395861
E-mail: azautne@gwdg.de

Running head: MSPP: A novel Method for typing below the species level

Supplementary Table 1. List of all possible amino acid substitutions and corresponding mass changes. Equal mass changes resulting in indistinguishable peak shifts are indicated in red

Substitution	ΔMW [Da]	Substitution	ΔMW [Da]	Substitution	ΔMW [Da]
I/L ←→ L/I	0.000	S ←→ C	16.061	G ←→ V	42.081
Q ←→ K	0.043	I/L ←→ M	18.033	I/L ←→ R	43.028
K ←→ E	0.941	H ←→ R	19.046	A ←→ D	44.010
I ←→ N	0.944	D ←→ H	22.053	C ←→ F	44.038
N ←→ D	0.985	N ←→ H	23.037	G ←→ C	46.087
Q ←→ E	0.985	L ←→ H	23.982	D ←→ Y	48.087
K ←→ M	3.018	M ←→ R	24.995	V ←→ F	48.044
P ←→ T	3.988	A ←→ P	26.038	N ←→ Y	49.072
Q ←→ H	9.010	H ←→ Y	26.035	C ←→ R	53.049
S ←→ P	10.039	S ←→ I/L	26.081	T ←→ R	55.082
T ←→ I	12.054	S ←→ N	27.026	A ←→ E	58.037
T ←→ N	12.999	T ←→ K	27.069	G ←→ D	58.037
V ←→ I/L	14.027	A ←→ V	28.054	P ←→ R	59.071
D ←→ E	14.027	K ←→ R	28.013	C ←→ Y	60.037
G ←→ A	14.027	Q ←→ R	28.057	S ←→ F	60.098
S ←→ T	14.027	A ←→ T	30.026	S ←→ R	69.109
N ←→ K	14.070	G ←→ S	30.026	G ←→ E	72.064
L ←→ Q	14.971	R ←→ W	30.026	L ←→ W	73.054
I ←→ K	15.015	T ←→ M	30.088	S ←→ Y	76.098
V ←→ D	15.956	P ←→ Q	31.014	C ←→ W	83.074
F ←→ Y	15.999	V ←→ M	32.060	G ←→ R	99.136
A ←→ S	15.999	I/L ←→ F	34.017	S ←→ W	99.135
P ←→ L	16.043	P ←→ H	40.024	G ←→ W	12.,161

Supplementary Table 3. Oligonucleotide primers used for sequencing of the *C. jejuni* genes included in the MSPP-scheme

ORF No. (NCTC 11168)	Gene	Forward primer (5'→3')	Reverse primer (5'→3')	Amplicon lenght [bp]
cj0095	rpmA	AAAGGTAGGTGCGCCTTTCG	CCACCATCGCCTGAAGCTAA	590
cj0155c	rpmE	AGGTCCTGAAGGTGGATTTAACA	TGGCATTCCTGCATCGCTTA	678
cj0244	rpmI	AGCAAAAGGTGTGCCAAGTT	ATGTCTGCGGCGTCTAACAA	447
cj0330c	rpmF	AGAAGTTGCTATCAGCGAGC	TCCAAAATCACCACCCATTGC	287
cj0370	rpsU	TACACAGAAAGGTGGTGAGGAC	TCCTTGAGCATTAGCCGCTT	490
cj0449c	-	TCACAGGCAAGGGTCCAATG	AGACTCTAAAGCCGCTTGTTCT	732
cj0450c	rpmB	ATCACGCAAGGTCCGAAAGT	GCATCTTTACCTTTTAGCTCTGACA	717
cj0471	rpmG	GCTTGATCGCTCCAGTAGCA	GAACCCCCAACCATCGGATT	380
cj0710	rpsP	AGCGTCGTATAGCAGAGGGA	AGAACTTGCATCCTTGCTCTT	665
cj0884	rpsO	AGCGTATTCGCTAGCTTTGGT	ATCGGCTATAACCGCTCTGC	658
cj0961c	rpmH	TGTTCCAGTTTTTCTTTCGTGC	GATCACACCTTCGCAATGCC	476
cj1591	rpmJ	GGAATTCATTCAAGGAGACGC	ACGGATAGCTGCTGCTTCAT	310
cj1611	rpsT	GGCGGACTAGGTGTAATGCT	GTTCCCCAAGTTCTGGATCGT	712
cj1694c	rpsN	GATGGTTTTGATGGACGCGG	AACACGACGACCAGGCTTAG	643

cj1696c	rplX	GGGGTAGCAAAAGAAGATACGC	CACCAGCACCTACGCTGATA	653
cj1698c	rpsQ	GCAGCTGAGCTTGCAACAAT	GCCCAAAGATACGAGTTCCG	717
cj1699c	rpmC	CTCACGCCAAATCGAAGCAG	TTCAACCAAAACACTTGCGGT	554
cj1703c	rpsS	CATCCAGTAACTCCGTGGGG	ACTGCGCTTGAAATTGCGTT	553
cj1705c	rplW	TGGCGCTTGAAAGAGCTTTAG	TGGCGCTTGAAAGAGCTTTAG	754

Supplementary Table 2. Overview of all isoforms included in the *C. jejuni* MSPP-scheme

Locus	Full name / product (ORF No. in NCTC 11168)	calc. Mass [Da]		Frequency in database
RpmA/BACT000056; L27-M; *cj0095*				
sequence	(M)AHKKGQGSTQNNRDSIGRRLGVKKFGGEFVRAGNIIIRQRGTATHAGNNVGMGKDHTIFALIDGFVKFERKDKDRKKVSVYPA (83 aa)			
1 *	11168 reference isoform	9153.45 Da	±0.00 Da	99.281% (3452/3477)
2	A47P	9179.48 Da	+26.03 Da	0.288% (10/3477)
3	M53T	9123.36 Da	-30.09 Da	0.201% (7/3477)
4	N49S	9126.42 Da	-27.03 Da	0.086% (3/3477)
5	V23I + N35D	9168.46 Da	+15.01 Da	0.056% (2/3477)
6	M1V	9121.39 Da	-32.06 Da	0.029% (1/3477)
7	I59L	9153.45 Da	±0.00 Da	0.029% (1/3477)
8	A84V	9181.50 Da	+28.05 Da	0.029% (1/3477)

RpmE/BACT000060; L31; *cj0155c*				
sequence	MKKEIHPEYVECKVSCACGNTFTTKSNKAELRVDICSNCHPFFTGSEKIVDAAGRVEKFKKKYAMQ (66 aa)			
1 *	11168 reference isoform	7476.68 Da	±0.00 Da	97.063% (3371/3473)
2	V10I	7490.71 Da	+14.03 Da	1.785% (62/3473)
3	T23A + Q66STOP	7318.52 Da	-158.16 Da	0.346% (12/3473)
4	T23A	7446.65 Da	-30.03 Da	0.259% (9/3473)
5	R32K	7448.67 Da	-28.01 Da	0.259% (9/3473)
6	T23I	7488.73 Da	+12.05 Da	0.086% (3/3473)
7	A53T	7506.71 Da	+30.03 Da	0.086% (3/3473)
8	G19E	7548.74 Da	+72.06 Da	0.058% (2/3473)
9	A64T	7506.71 Da	+30.03 Da	0.029% (1/3473)
10	V10I + S15N + T23I + R32K + A53T + K61R + A64S	7575.81 Da	+99.13 Da	0.029% (1/3473)

RpmI/BACT000064; L35; *cj0244*				
sequence	MPKMKSVKSAVKRFKVGKNKIKRGSAFRSHILTKKPAKRMRGLRTAKYVHSTNVKAVEKMLGI (63 aa)			
1 *	11168 reference isoform	7153.78 Da	±0.00 Da	25.137% (874/3477)
2 *	G42D	7211.81 Da	+58.03 Da	71.412% (2483/3477)
3	G42D + A46T	7241.84 Da	+88.06 Da	2.215% (77/3477)
4	G42N	7210.83 Da	+57.05 Da	1.064% (37/3477)
5	P2S + G42D	7201.77 Da	+47.99 Da	0.058% (2/3477)
6	G42D + K55E + E58K	7211.81 Da	+58.03 Da	0.029% (1/3477)
7	A26S + G42D	7227.81 Da	+74.03 Da	0.029% (1/3477)
8	G42D + S51N	7238.84 Da	+85.06 Da	0.029% (1/3477)
9	A10T + G42D	7241.84 Da	+88.06 Da	0.029% (1/3477)

RpmF/BACT000061; L32-M; *cj0330c*				
sequence	(M)AVPKRRVSKTRAAKRRTHYKVSLPMPIKDKDGSYKMPHRANPTTKEY (47 aa)			
1 *	11168 reference isoform	5496.45 Da	±0.00 Da	92.955% (3232/3477)
2 *	I28V	5510.48 Da	+14.03 Da	3.365% (117/3477)
3 *	M26I	5514.48 Da	+18.03 Da	2.100% (73/3477)
4 *	S23N	5523.48 Da	+27.03 Da	0.661% (21/3477)
5	P43L	5512.49 Da	+16.04 Da	0.460% (16/3477)
6	M26T	5466.36 Da	-30.09 Da	0.115% (4/3477)
7	A13T	5526.48 Da	+30.03 Da	0.115% (4/3477)
8	P38S	5486.41 Da	-10.04 Da	0.058% (2/3477)
9	R40C	5443.40 Da	-53.05 Da	0.029% (1/3477)
10	V3G	5454.37 Da	-42.08 Da	0.029% (1/3477)
11	R40H	5477.40 Da	-19.05 Da	0.029% (1/3477)
12	M37I	5478.42 Da	-18.03 Da	0.029% (1/3477)
13	T44N	5483.45 Da	-13.00 Da	0.029% (1/3477)
14	V22I + M37I + T44V	5490.47 Da	-5.98 Da	0.029% (1/3477)
15	I28V + S34N	5509.45 Da	+13.00 Da	0.029% (1/3477)
16	A13V + I28V	5510.48 Da	+14.03 Da	0.029% (1/3477)

RpsU/BACT000021; S21; *cj0370*				
sequence	VPGIKVHPNESFDEAYRKFKKQVDRNLVVTEVRARRFFEPMTEIRKKQKISARKKMLKRLYMLRRYESRL (70 aa)			
1 *	11168 reference isoform	8641.27 Da	±0.00 Da	99.827% (3469/3475)
2	N9S	8614.24 Da	-27.03 Da	0.029% (1/3475)
3	M56I	8623.23 Da	-18.04 Da	0.029% (1/3475)
4	P8S	8631.23 Da	-10.04 Da	0.029% (1/3475)
5	V23L	8655.29 Da	+14.02 Da	0.029% (1/3475)
6	P2L	8657.31 Da	+16.04 Da	0.029% (1/3475)
7	A15V	8669.32 Da	+28.05 Da	0.029% (1/3475)

Cj0449c (protein of unknown function)				
sequence	MLHEYRELMSELKGKDAHFDKLFDRHNELDDMIKDAEEGRTSLSSMEISTLKKEKLHVKDELSQYLANYKK (71 aa)			
1 *	11168 reference isoform	8458.60 Da	±0.00 Da	97.462% (2304/2364)
2 *	Q64R	8486.65 Da	+28.05 Da	0.465% (11/2364)
3	D24E + M32Q + T41N + S42L + S45D + M46I + T50N + V58I + E61Q + S63N	8571.69 Da	+113.09 Da	0.423% (10/2364)
4	V58I	8472.62 Da	+14.02 Da	0.381% (9/2364)
5	A67V	8486.65 Da	+28.05 Da	0.169% (4/2364)
6	D24Q + M32Q + S42P + S45D + M46L + T50S + S63N	8501.59 Da	+42.99 Da	0.169% (4/2364)
7 *	K15R + V58I	8500.64 Da	+42.04 Da	0.123% (3/2364)
8	D24E + M32Q + T41A + S42L + S45D + M46I + T50N + V58I + S63N + A67S	8545.65 Da	+87.05 Da	0.123% (3/2364)
9	S49L	8484.68 Da	+26.08 Da	0.085% (2/2364)
10	E11K + Q64R	8485.71 Da	+27.11 Da	0.085% (2/2364)
11	A36V	8486.65 Da	+28.05 Da	0.085% (2/2364)
12	E7K + S45G	8427.63 Da	-30.97 Da	0.042% (1/2364)
13	V58A	8430.54 Da	-28.06 Da	0.042% (1/2364)
14	T41S	8444.57 Da	-14.03 Da	0.042% (1/2364)
15	M9I + V58I	8454.59 Da	-4.01 Da	0.042% (1/2364)
16	D16N	8457.61 Da	-0.99 Da	0.042% (1/2364)
17	D20N	8457.61 Da	-0.99 Da	0.042% (1/2364)
18	E11K + S45N	8484.68 Da	+26.08 Da	0.042% (1/2364)
19	A17T	8488.62 Da	+30.02 Da	0.042% (1/2364)
20	G39D	8516.63 Da	+58.03 Da	0.042% (1/2364)
21	S45N + S63N + A67T	8542.68 Da	+84.08 Da	0.042% (1/2364)
22 *	S44N + Q64R	8513.68 Da	+55.08 Da	0.000% (0/2364)

RpmB/BACT000057; L28-M; *cj0450c*				
sequence	(M)ARVCQITGKGPMVGNNVSHANNKTKRRFLPNLRTVRVTLEDGTTRKMRIAASTLRTLKKQNSK* (63 aa)			
1 *	11168 reference isoform	7064.27 Da	±0.00 Da	98.446% (3421/3475)
2	P12S + T44A	7024.20 Da	-40.07 Da	0.547% (19/3475)
3	V4I + R46K	7050.21 Da	-13.99 Da	0.460% (16/3475)
4	V38I	7078.22 Da	+14.02 Da	0.177% (6/3475)
5	V4I	7078.22 Da	+14.02 Da	0.144% (5/3475)
6	T8A	7034.17 Da	-30.03 Da	0.086% (3/3475)
7	R3K + P12L	7052.23 Da	-11.97 Da	0.058% (2/3475)
8	A51T	7094.22 Da	+30.02 Da	0.058% (2/3475)
9	V4I + V38I + T44S + T45I + R49K	7062.26 Da	-1.94 Da	0.029% (1/3475)

RpmG/BACT000062; L33; *cj0471*				
sequence	MRIKVGLKCEECGDINYSTYKNSKNTTEKLELKKYCPRLKKHTLHKEVKLKS (52 aa)			
1 *	11168 reference isoform	6156.29 Da	±0.00 Da	99.281% (3452/3477)
2	E28V	6126.31 Da	-29.98 Da	0.259% (9/3475)
3	K41R	6184.31 Da	+28.02 Da	0.144% (5/3475)
4	E28V+ S52N	6153.33 Da	-2.96 Da	0.086% (3/3475)
5	T43I	6168.35 Da	+12.06 Da	0.086% (3/3475)
6	L44F	6190.31 Da	+34.02 Da	0.058% (2/3475)
7	R2K	6128.28 Da	-28.01 Da	0.029% (1/3475)
8	R38K	6128.28 Da	-28.01 Da	0.029% (1/3475)
9	T19I	6168.35 Da	+12.06 Da	0.029% (1/3475)

RpsP/BACT000016; S16; *cj0710*				
sequence	MTVIRLTRMGRTKRPFYRIVVTDSRKRRDGGWIESIGYYNPMVEPEVIKVDAERLAYWKSVGAKLSDKVASITSK* (75 aa)			
1 *	11168 reference isoform	8678.14 Da	±0.00 Da	98.159% (3412/3476)
2 *	E53K	8677.20 Da	-0.94 Da	1.093% (38/3475)
3 *	A63V	8706.20 Da	+28.06 Da	0.230% (8/3475)
4	A56T	8708.17 Da	+30.03 Da	0.086% (3/3475)
5	A70T	8708.17 Da	+30.03 Da	0.086% (3/3475)
6	R27C	8625.09 Da	-53.05 Da	0.058% (2/3475)
7	V43I	8692.17 Da	+14.03 Da	0.058% (2/3475)
8	D51N + A52T	8707.18 Da	+29.04 Da	0.058% (2/3475)
9	Y38H	8652.11 Da	-26.03 Da;	0.029% (1/3475)
10	D29N	8677.16 Da	-0.98 Da	0.029% (1/3475)
11	A52S	8694.14 Da	+16.00 Da	0.029% (1/3475)
12	A52V	8706.20 Da	+28.06 Da	0.029% (1/3475)
13	A52T	8708.17 Da	+30.03 Da	0.029% (1/3475)
14	G31S + V47L + A52I + A56N	8807.30 Da	+129.16 Da	0.029% (1/3475)

RpsO/BACT000015; S15-M; *cj0884*				
sequence	(M)ALDSAKKAEIVAKFAKKPGDTGSTEVQVALLTARIAELTEHLKIYKKDFSSRLGLLKLVGQRKRLLSYLKRKDYNSYSKLITELNLRDK (89 aa)			
1 *	11168 reference isoform	10094.85 Da	±0.00 Da	93.327% (3245/3477)
2 *	E10A	10036.82 Da	-58.03 Da	3.020% (105/3477)
3	A37T	10124.88 Da	+30.03 Da	2.962% (103/3477)
4	A37I+ N76D	10137.92 Da	+43.07 Da	0.345% (12/3477)
5	V29I	10108.88 Da	+14.03 Da	0.086% (3/3477)
6	S79G	10064.83 Da	-30.02 Da	0.029% (1/3477)
7	P19A	10068.82 Da	-26.03 Da	0.029% (1/3477)
8	E10Q + V29I + A37I + E38D + N76S + T83A	10078.90 Da	-15.95 Da	0.029% (1/3477)
9	A9S	10110.85 Da	+16.00 Da	0.029% (1/3477)
10	A34V	10122.91 Da	+28.06 Da	0.029% (1/3477)
11	P19S+ A37I + N76D	10127.88 Da	+33.03 Da	0.029% (1/3477)
12	E10K + A37I	10135.99 Da	+41.14 Da	0.029% (1/3477)
13	A37I	10136.94 Da	+42.09 Da	0.029% (1/3477)

RpmH/BACT000063; L34 ;cj0961				
sequence	MKRTYQPHGTPRKRTHGFRVRMKTKNGRKVINARRAKGRKRLAV (44 aa)			
1 *	11168 reference isoform	5244.28 Da	±0.00 Da	99.655% (3463/3475)
2	R14S	5175.17 Da	-69.11 Da	0.086% (3/3475)
3	R39K	5216.27 Da	-28.01 Da	0.058% (2/3475)
4	Q6E	5245.27 Da	+0.99 Da	0.058% (2/3475)
5	G9S	5274.31 Da	+30.03 Da	0.058% (2/3475)
6	P7S	5234.24 Da	-10.04 Da	0.029% (1/3475)
7	R28H	5225.24 Da	-19.04 Da	0.029% (1/3475)
8	V20M + V30I + A33T	5320.39 Da	+76.11 Da	0.029% (1/3475)

RpmJ/BACT000065; L36; *cj1591*				
sequence	MKVRPSVKKMCDKCKVVRRKGVVRIICENPKHKQRQG (aa 37)			
1 *	11168 reference isoform	4364.39 Da	±0.00 Da	99.367% (3455/3477)
2	V23I	4378.41 Da	+14.02 Da	0.633% (22/3477)
3	V16I+ V22I	4392.44 Da	+28.05 Da	0.029% (1/3477)

RpsT/BACT000020; S20-M; *cj1611*				
sequence	(M)ANHKSAEKRARQTIKKTERNRFYRTRLKNITKAVREAAANGNKNAANEALKVANKSIHAMVSRGFIKKQTASRRVSRLALLVNKIA (86 aa)			
1 *	11168 reference isoform	9685.29 Da	±0.00 Da	38.982% (1355/3476)
2 *	N43D	9686.28 Da	+0.99 Da	60.184% (2092/3476)
3	N43D + R64H	9667.23 Da	-18.06 Da	0.403% (14/3476)
4	V35I + N43D	9700.30 Da	+15.01 Da	0.086% (3/3476)
5	G42E + N43D	9758.34 Da	+73.05 Da	0.086% (3/3476)
6	A34T + N43D	9716.30 Da	+31.01 Da	0.058% (2/3476)
7	N43D + A80T	9716.30 Da	+31.01 Da	0.058% (2/3476)
8	R36K + N43D	9658.26 Da	-27.03 Da	0.029% (1/3476)
9	A39V + N43D + R64H	9695.28 Da	+9.99 Da	0.029% (1/3476)
10	T14I + N43D	9698.33 Da	+13.04 Da	0.029% (1/3476)
11	I31L + N43D + A46T + V53I + I67L	9730.33 Da	+45.04 Da	0.029% (1/3476)
12	G42R + N43D	9785.41 Da	+100.12 Da	0.029% (1/3476)

RpsN/BACT000014; S14-M; *cj1694c*				
sequence	(M)AKKSMIAKAARKPKFKVRAYTRCQICGRPHSVYRDFGICRVCLRKMGNEGLIPGLKKASW (60 aa)			
1 *	11168 reference isoform	6825.27 Da	±0.00 Da	96.520% (3356/3477)
2 *	A20G	6811.24 Da	-14.03 Da	3.135% (109/3477);
3	T22A	6795.25 Da	30.02 Da	0.230% (8/3477)
4	P14A	6799.23 Da	-26.04 Da	0.029% (1/3477)
5	V18I	6839.30 Da	+14.03 Da	0.029% (1/3477)
6	G38E	6897.34 Da	+72.07 Da	0.029% (1/3477)
7 [a]	M1V + A10N + A11K + K13P + P14A + F16H + K17S + V18T + R19Q + Q25E + I26R + D36K + G38H + I39L + V42I + L44F + K46E + M47L + G48A + N49Y + E50K + L52Q + L56V	6990.26 Da	+164.99 Da	0.029% (1/3477)

RplX/BACT000053; L24-M; *cj1696*				
sequence	(M)AVKLKIKKGDSVKVITGDDKGKTGKVLAVYPKTLKVVVEGCKIAKKAIKPSEKNPNGGFINKEMPMDISNVAKVQE (76 aa)			
1 *	11168 reference isoform	8151.75 Da	±0.00 Da	99.280% (3452/3477)
2	S12N	8178.78 Da	+27.03 Da	0.374% (13/3477)
3	A73V	8179.80 Da	+28.05 Da	0.115% (4/3477)
4	S12N + I16V	8164.75 Da	+13.00 Da	0.086% (3/3477)
5	T24I + P56S	8153.77 Da	+2.02 Da	0.058% (2/3477)
6	P51S	8141.71 Da	-10.04 Da	0.029% (1/3477)
7	A48T	8181.78 Da	+30.03 Da	0.029% (1/3477)
8	V3L + S12N + V38L + E40Q +V75I	8219.87 Da	+68.12 Da	0.029% (1/3477)

RpsQ/BACT000017; S17; *cj1698*				
sequence	MAFKREIQGVVVKIAGEKTASVLVERKVVHPRYRKIVKRFKKYLIHDERNEVKVGDTVVAVECRPLSKRKSFRLKSVLATGVE (83 aa)			
1 *	11168 reference isoform	9549.39 Da	±0.00 Da	99.482% (3459/3477)
2	A20V+ R32K	9549.43 Da	+0.04 Da	0.460% (16/3477
3	V24I	9563.42 Da	+14.03 Da	0.029% (1/3477)
4	A20V + V22I + V58I	9605.50 Da	+56.11 Da	0.029% (1/3477)

RpmC/BACT000058; L29; *cj1699*				
sequence	MKYTEIKDKTAAELATMLKEKKVLLFTLKQKLKTMQLTNPKEISQVKKDIARINTAINALR (61 aa)			
1 *	11168 reference isoform	7033.49 Da	±0.00 Da	99.799% (3470/3477)
2	A12V	7061.55 Da	+28.06 Da	0.144% (5/3477)
3	T38A	7003.47 Da	-30.02 Da	0.029% (1/3477);
4	K47R + R61K	7033.49 Da	±0.00 Da	0.029% (1/3477)

RpsS/BACT000019; S19-M; *cj1703c*				
sequence	(M)ARSLKKGPFVDDHVMKKVIAAKKANDNKPIKTWSRRSTITPDMIGLTFNVHNGKSFIPVYITENHIGYKLGEFAPTRTFKGHKGSVQKKIGK (92 aa)			
1 *	11168 reference isoform	10322.08 Da	±0.00 Da	97.929% (3405/3477)
2 *	K24E	10323.02 Da	+0.94 Da	1.726% (60/3477)
3	P76Q	10353.09 Da	+31.01 Da	0.086% (3/3477);
4	S56N	10349.11 Da	+27.03 Da	0.058% (2/3477)
5	T47A	10292.05 Da	-30.03 Da	0.029% (1/3477)
6	V15A	10294.03 Da	-28.05 Da	0.029% (1/3477)
7	M16I	10304.05 Da	-18.03 Da	0.029% (1/3477)
8	T41I	10334.13 Da	+12.05 Da	0.029% (1/3477)
9	H52Y	10348.12 Da	+26.04 Da	0.029% (1/3477)
10	T41I + A75S	10350.13 Da	+28.05 Da	0.029% (1/3477)
11	A2T	10352.11 Da	+30.03 Da	0.029% (1/3477)

RplW/BACT000052; L23; *cj1705c*				
sequence	MADITDIKTILYTEKSLNLQEQGVVVIQTSPKMTKTGLKAVLKEYFGVTPKSINSLRMDGKIKRFRGRLGQRNNYKKFYVKLPEGVSLENTEA (93 aa)			
1 *	11168 reference isoform	10567.35 Da	±0.00 Da	49.640% (1726/3477)
2	I62V + N74D	10554.31 Da	-13.04 Da	37.388% (1300/3477)
3	A40V	10595.41 Da	+28.06 Da	9.117% (317/3477)
4	I62V + N74D + T91A	10524.29 Da	-43.06 Da	2.013% (70/3477)
5	T91M	10597.44 Da	+30.09 Da	0.863% (30/3477)
6	N74D	10568.34 Da	+0.99 Da	0.633% (22/3477)
7	V25L + I62V + N74D + T91A	10538.31 Da	-29.04 Da	0.230% (8/3477)
8	I62V + N74D + N90S + T91M	10557.37 Da	-9.98 Da	0.029% (1/3477)
9	E84Q	10566.37 Da	-0.98 Da	0.029% (1/3477)
10	I62V + N74D + V80I	10568.34 Da	+0.99 Da	0.029% (1/3477)
11	A40V + STOP94Q+Ins 95D+ 96G + 97N	11009.78 Da	+442.43 Da	0.029% (1/3477)
12 *	I62V + N74D + N90D + T91A	10525.27 Da	-42.08 Da	0.000% (0/3477)
13 *	I62V + N74D + N90D	10555.30 Da	-12.05 Da	0.000% (0/3477)
14 *	A40V + N90D	10596.39 Da	+29.04 Da	0.000% (0/3477)

Legend:
* observed in test population
AA numbering including start-methionin, if mass spectrometry indicates its absence it is written in brackets (M)

Supplementary Figure 1A

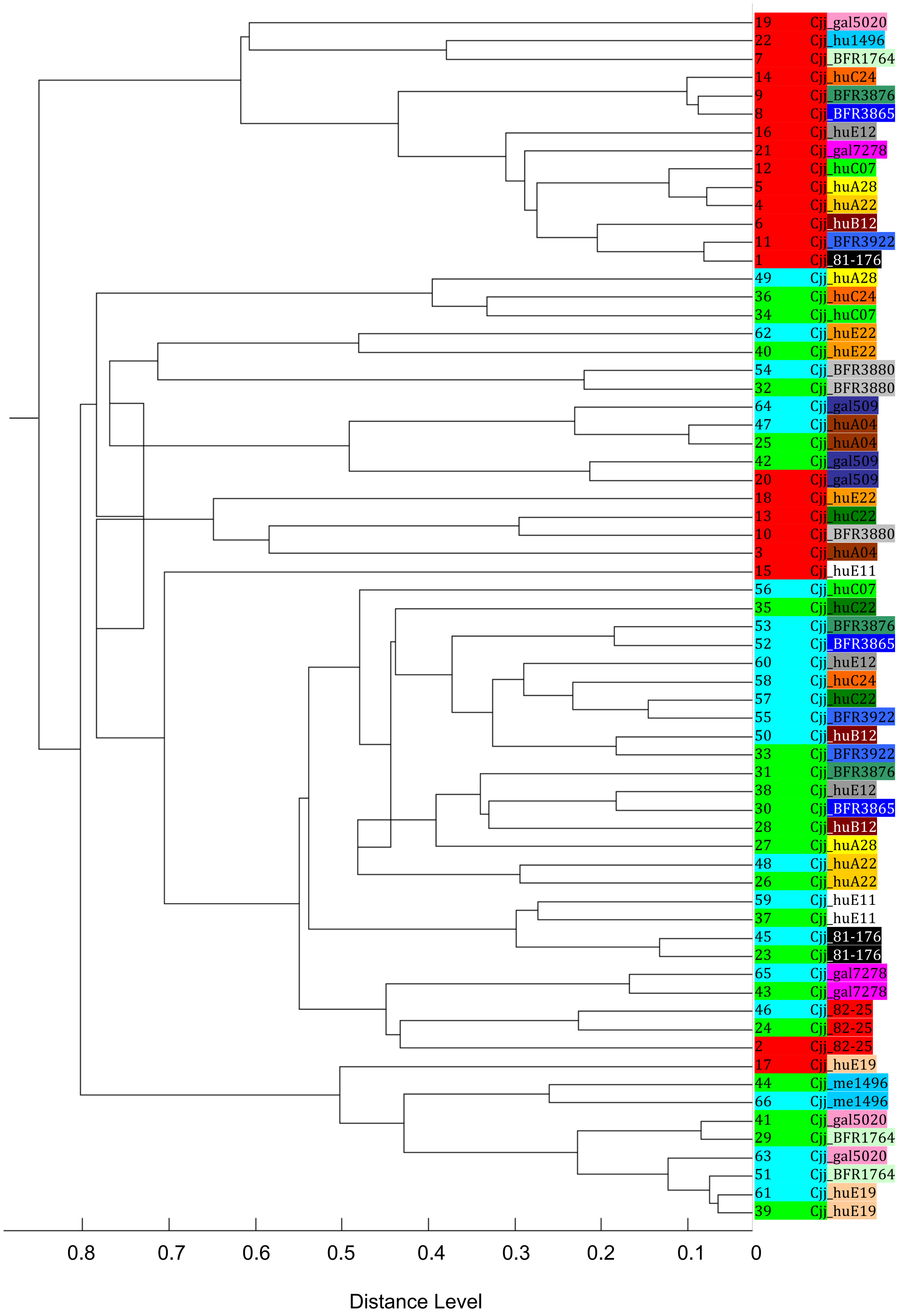

Supplementary Figure 1B

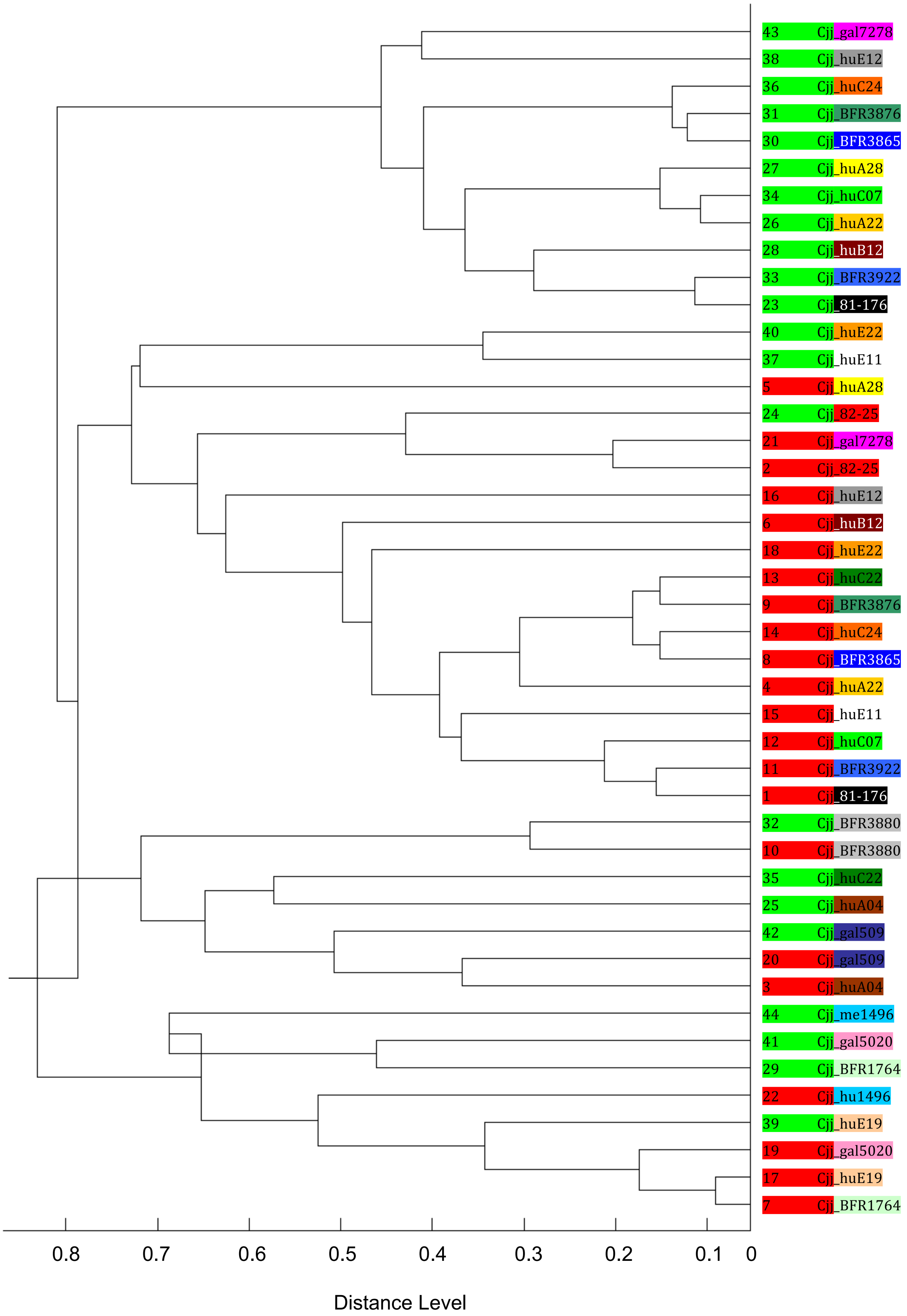

Supplementary Figure 1C

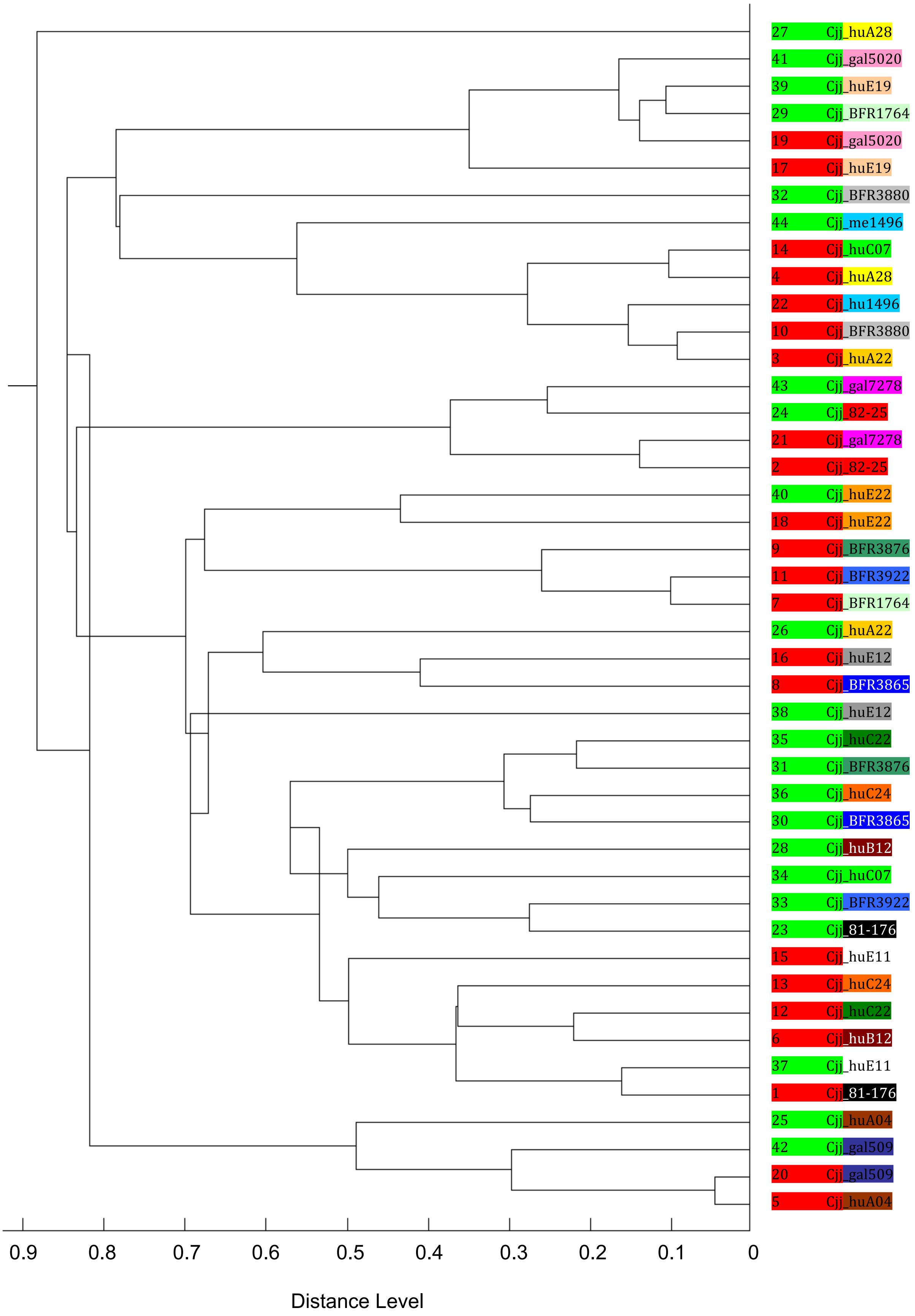

Supplementary Figure 2

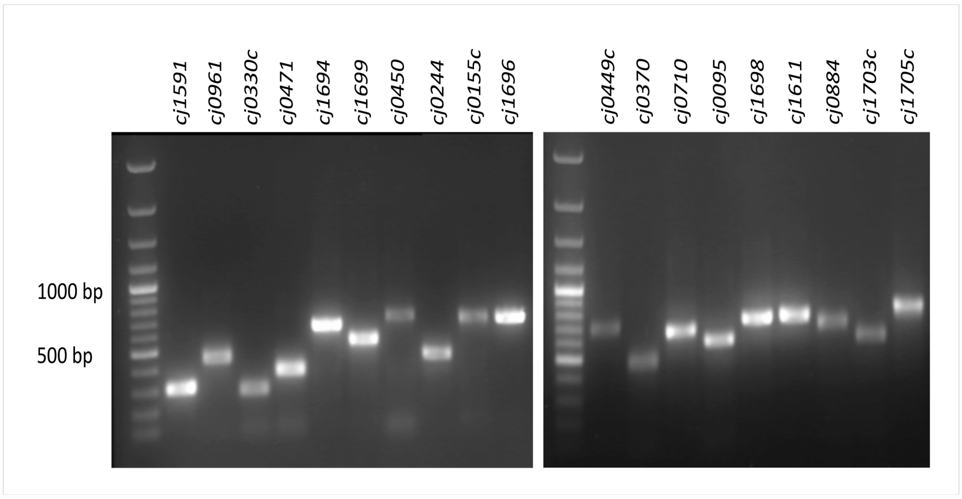

SUPPLEMENTARY TABLES & FIGURES LEGENDS

SUPPLEMENTARY TABLES

Supplementary Table 1. List of possible amino acid substitutions and corresponding calculated mass changes. Equal mass changes that resulted in indistinguishable peak shifts are indicated in red.

Supplementary Table 2. Overview of all isoforms included in the *C. jejuni* ssp. *jejuni* MSPP-scheme. An asterisk indicates each isoform detected in the MSPP-typed *C. jejuni* ssp. *jejuni* isolate collection. Isoform no. 22 of Cj449c and isoforms no. 12, 13, and 14 of Cj1705c had not been deposited in the *C. jejuni* ssp. *jejuni* wgMLST database and rMLST database respectively at the time the analysis was performed. But they were detected in the present study for the fist time. As a result, their frequency is given as 0.0%.

Supplementary Table 3. Oligonucleotides. Oligonucleotide primers used for amplification and sequencing of the *C. jejuni* ssp. *jejuni* genes included in the MSPP-scheme.

SUPPLEMENTARY FIGURES

Supplementary Figures 1 A-C. ICMS-spectra-based PCA-dendrograms of 22 *C. jejuni* ssp. *jejuni* isolates cultured under different culture conditions

22 *C. jejuni* spp. *jejuni* isolates representing the 22 different MSPP sequence types have been cultured for 24 hrs on **(A)** Columbia sheep blood agar plates (COS, red), Mueller Hinton horse blood agar (MHF, green), and Oxoid Campylobacter agar supplemented with lysed horse blood (CAM, blue) at 42°C; on **(B)** 2 different batches of Columbia sheep blood agar plates (different lots, lot A red, lot B green) each at 42°C; and on **(C)** 2 different batches of Columbia sheep blood agar plates (same lot) at 42°C (red) and 37°C (green). The mass spectra of all 110 samples have been recorded and PCA cluster analyses performed. Each of the 22 bacterial isolates is indicated by a specific color (shading of the isolate designation). According to the phylogenetic identity it has been expected that independent of the different culture conditions identical isolates group as couples or triplets.

A: Instead of the grouping as triplets, isolates cultured on COS agar plates (red) group in two clusters. Two smaller subclusters are formed exclusively by isolates cultured on MHF agar (green) and CAM agar (blue). Only 13 isolates group into pairs MHF associated with CAM agar culture. This can be explained with the fact that both CAM and MHF agar are supplemented with horse blood.

B: Bacterial isolates group into 3 major clusters. The upper consists completely of isolates cultured on lot B COS agar plates (green). The middle cluster consists of 15 (83.3%) isolates cultured on lot A COS agar plates and only 3 isolates cultured on lot B COS agar plates. Only the third lower cluster is of mixed composition (lot A and B) containing 7 isolates that can be matched.

C: *C. jejuni* culture on the same COS agar plate lot but at different temperatures, 42°C (red) and 37°C (green), results in pairwise clustering (taking all degrees of freedom into account) only in the case of 11 isolates (50%) in contrast the remaining isolates form smaller subclusters according correlating with the incubation temperature.

In summary, the different culture conditions significantly interfered with the phylogenetic identity.

Supplementary Figure 2. Amplicons obtained by PCR of the 19 genes corresponding to the biomarker ions. Oligonucleotide primer pairs (listed in Supplementary Table 3) resulted in specific, single bands of the predicted size.
